# Beyond diagnosis: exploring the extended clinical utility of urine fluorescence in situ hybridization in upper tract urothelial carcinoma

**DOI:** 10.1186/s12894-025-02025-w

**Published:** 2025-12-22

**Authors:** Yihao Zhao, Nianzhao Zhang, Shijie Zhang, Xiaoyi Zhang, Lei Liu, Jun Chen

**Affiliations:** 1 Department of Urology, Qilu Hospital of Shandong University, Jinan, Shandong China; 2 Department of Medicine, Jacobi Medical Center, Albert Einstein College of Medicine, The Bronx, NY USA

**Keywords:** Upper tract urothelial carcinoma, Fluorescence in situ hybridisation, Risk stratification, Differential diagnosis, Tumor staging

## Abstract

**Background:**

While urine fluorescence in situ hybridization (FISH) has established itself as a crucial diagnostic modality for upper tract urothelial carcinoma (UTUC), the broader clinical applications of this technique are yet to be fully elucidated.

**Methods:**

This retrospective cohort study analyzed patients who underwent urine FISH test at Qilu Hospital of Shandong University from 2022 to 2025. We assessed its clinical value by constructing ROC curves. Additionally, a novel predictive tool based on urine FISH features and blood routine examination indicators was then developed to enhance urine FISH’s staging predictive ability. We also explored the correlation of urine FISH features with LVI, Ki67, and HER2.

**Results:**

The study encompassed 220 patients, revealing the utility of urine FISH test in informing clinical treatment. Particularly, among the urine FISH features evaluated, CEP7 demonstrated superior performance in identifying patients requiring radical nephroureterectomy (AUC = 0.885), distinguishing renal pelvis carcinoma (AUC = 0.926), and discriminating non-UTUC individuals (AUC = 0.911). Regrettably, urine FISH features demonstrated constrained predictive efficacy in UTUC staging. Consequently, leveraging machine learning, we developed a staging prediction tool based on urine FISH features and blood routine examination indicators, with the SVM model demonstrating optimal predictive performance. In addition, we observed that certain urine FISH features were associated with the expression levels of HER2 and Ki67 in neoplastic tissues.

**Conclusions:**

Our study provides an initial exploration into the extended utility of urine FISH beyond conventional diagnostics in UTUC, thereby offering novel insights for the advancement of precision oncology in this disease.

**Clinical trial number:**

Not applicable.

**Retrospectively registered:**

KYLL-202412-044.

**Supplementary Information:**

The online version contains supplementary material available at 10.1186/s12894-025-02025-w.

## Background

Upper tract urothelial carcinoma (UTUC) is a malignant tumor originating from the uroepithelium of the renal pelvis or ureter and accounts for 5–10% of all urothelial carcinoma [1]. Radical nephroureterectomy (RUN) is the standard treatment for UTUC, but the limitations of this surgical approach to trauma cannot be ignored [2]. For low-risk UTUC or benign upper urinary tract lesions, the American Urological Association (AUA) guideline suggests that kidney-sparing surgery (KSS) can be performed in order to ensure the effectiveness of lesion treatment while preserving the renal function of the patient as much as possible [3]. For patients with locally advanced UTUC (pT_2 − 4_/N+), surgery alone is often ineffective. In recent years, scholars have begun to pay attention to the value of neoadjuvant chemotherapy (NAC) for these patients [4–6]. However, there is a lack of accurate risk stratification and staging tools in clinical practice, making it difficult for patients to escape from the“one-size-fits-all” dilemma in the actual treatment process [7]. In addition, the differential diagnosis of renal pelvis cancer (RPC) is also difficult. Some renal cell carcinoma (RCC) could grow into the renal sinus and invade the renal pelvis, which can be easily confused with RPC [8].

Although imaging, ureteroscopy, and urine cytology are important assessment tools to assist surgeons in making clinical decisions about patients with UTUC, these methods have limitations in clinical practice. Computed tomography urography (CTU) can effectively diagnose UTUC, but its ability to evaluate pathological staging is insufficient [9]. Ureteroscopy can visualize the lesion site and obtain the pathology of the mass, however, studies have shown that 89.6% of patients with clinical stage ≤ cT1 have muscle invasion in the final pathology [10, 11]. Urine cytology is a simple test that determines the presence of tumors by analyzing the morphology of exfoliated cells in the urine [12]. Nevertheless, the sensitivity of the results is extremely low and is easily interfered with by many factors [13]. Hence, there is a pressing need for innovative markers to further advance precision medicine for UTUC.

Fluorescence in situ hybridization (FISH) is a specific and non-invasive screening approach for UTUC. It predicts tumorigenesis based on cellular chromosomal aberrations and has demonstrated outstanding clinical sensitivity and specificity [14]. Previous studies have shown that urine FISH features are associated with tumor staging and grading [15]. In light of this, our study aimed to further investigate the extended application value of this technique, with the objective of providing novel clinical insights for the management of this disease.

## Methods

### Patient population

Patients who underwent urine FISH test in Qilu Hospital of Shandong University during outpatient consultation or hospitalization from August 2022 to April 2025 were taken as the study subjects (approval number: KYLL-202412-044), and the patients’ urine FISH test, imaging data, and pathological results were collected and analyzed in this retrospective study. Inclusion criteria: (1) Patients underwent urinary exfoliative cell FISH test within 2 months before surgery; (2) Patients underwent imaging examination within 2 months before surgery; (3) The lesions were located in the upper urinary tract; (4) The lesions were histopathologically examined to obtain a clear pathologic diagnosis.

### Urine FISH test and interpretation of results

Collect morning urine from patients and send it to the hospital for FISH test within 2 h. Urine FISH test was performed using FISH kit (HealthCare, Wuhan). The urine FISH results were determined by assessing Chromosome Enumerating Probe 3/7/17 (CEP3/7/17) and P16 Probe (P16) (Fig. S1). The detailed determination criteria were recorded in Additional Sect. 1.

### Extended application value of urine FISH

To explore the role of urine FISH in surgical decision-making for upper urinary tract lesions, we divided patients into RUN group (patients with high-risk UTUC) and KSS group (patients with low-risk UTUC or benign upper urinary tract lesions) based on whether nephron-sparing was performed intraoperatively. To evaluate the diagnostic capability of urine FISH in distinguishing RPC, we categorized patients suffering from malignant tumors of the renal pelvic region into RPC group and RCC group according to pathological findings. To evaluate the predictive value of tumor staging for UTUC, patients were categorized into non-muscle-invasive upper tract urothelial carcinoma (NMIUTUC) group and muscle-invasive upper tract urothelial carcinoma (MIUTUC) group based on tumor T staging results. Additionally, we dichotomized the patients into UTUC and non-UTUC groups to corroborate the diagnostic accuracy of urine FISH for UTUC. The detailed grouping criteria were recorded in Additional Sect. 1.

### Further screening for UTUC patients

To construct a staging prediction model, we further screened UTUC patients. Inclusive criteria: (1) Pathological examination can clarify whether the tumor invades the muscle layer or not; (2) Available medical data (missing data < 30%). Exclusion criteria: (1) History of comorbidities with other primary tumors; (2) Patients with any chronic inflammatory diseases; (3) Patients using myelosuppressive agents before surgery. Patient information, including blood routine examination within 2 weeks preoperatively, was retrieved from electronic medical records. Based on these data, the inflammation indicators were calculated. All predictor variables included in the study were listed in Table S1.

### Preliminary development of a CEP17-based staging prediction tool

The screened UTUC patients were randomly assigned to the training and test sets according to 7:3. Variables were selected using the Mann-Whitney U test or T test within the training set. Z-score standardization was performed on the variables to improve data comparability and model stability.

We selected 8 commonly used machine learning algorithms (ML), including decision tree (DT), random forest (RF), support vector machine (SVM), xgboost (XGB), elastic net (Enet), logistic regression (LR), light gradient boosting machine (LGBM) and k-nearest neighbours (KNN). The R package “tidymodels” was used for model construction, and the optimal parameters for each algorithm were selected using grid tuning on the training set. All ML models were validated on the test set. We chose the model with the highest area under the curve (AUC) of the subject’s operating characteristics as the optimal model. Decision curve analysis (DCA) was performed to graphically evaluate the clinical net benefit of the models across a range of probability thresholds. The contribution and influence of each feature to the optimal model was accurately calculated using the SHAP method.

### Statistical analysis

The counting data was reported as quantity (percentage), and the patients’ age was converted into two categorical variable with 65 years as the critical value. The differences in clinical pathological information between the NMIUTUC patients and MIUTUC patients were compared using Pearson’s chi square test. The missing data was addressed through mean imputation. The diagnostic performance of the model was evaluated using receiver operating characteristic (ROC) curve analysis, with metrics including the AUC, sensitivity, specificity, positive predictive value (PPV), negative predictive value (NPV), recall and precision. Spearman correlation analysis was used to calculate the correlation of lymphovascular invasion (LVI), Ki67 and HER2 with urine FISH features. All data were analyzed using R4.4.0 software. Statistical significance was defined as *P* < 0.05.

## Results

### Basic patient information and study flow

**Fig. 1:**
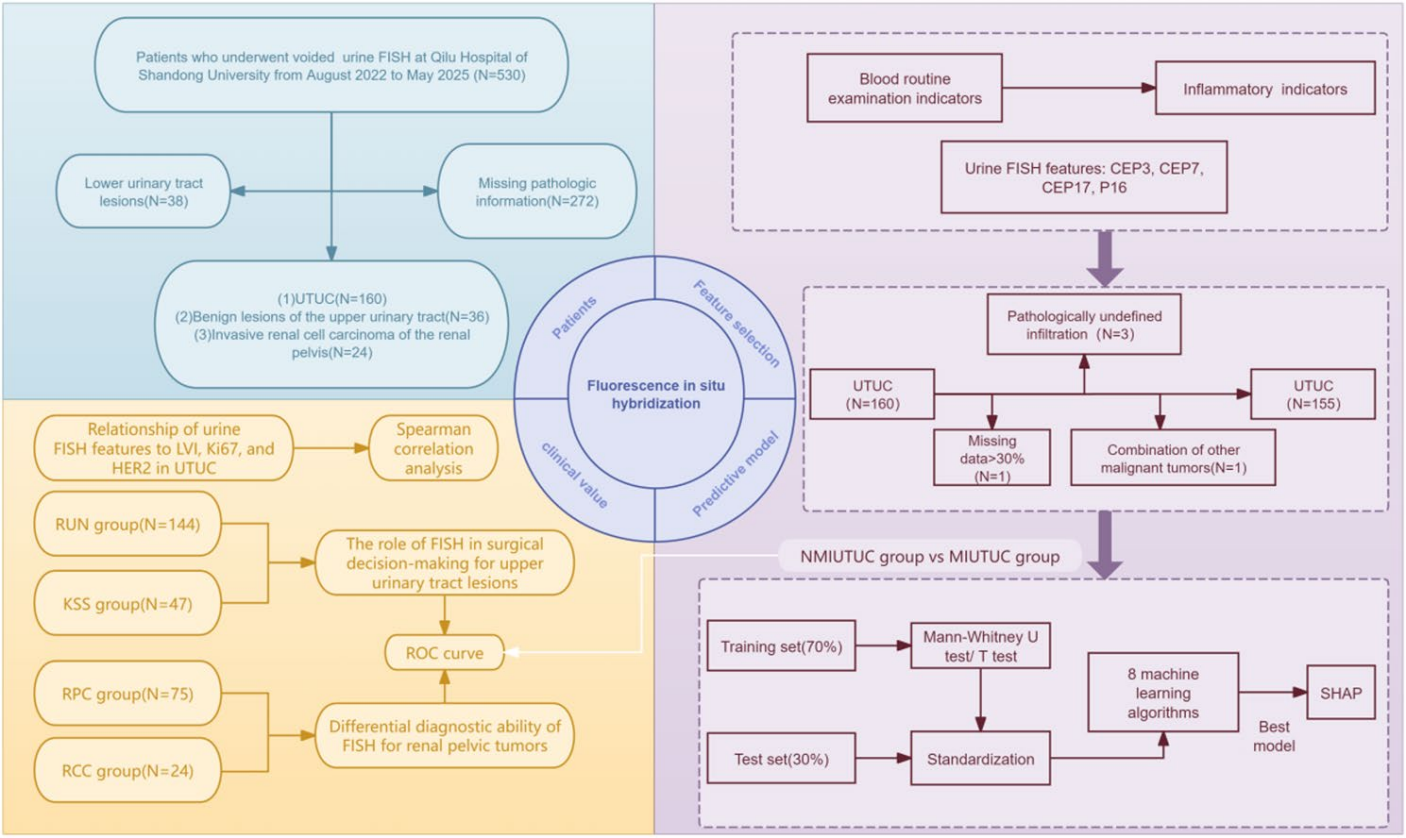
Outline of the analyses performed in this study. The blue module represents patient screening and subgroup stratification; the yellow module represents the clinical value of urine FISH for UTUC; the purple module represents the process of UTUC staging prediction model development and validation

A total of 220 patients were enrolled in this study, among whom 5 had incomplete clinical data (extent of missing data < 30%). Among them, 160 were pathologically diagnosed with UTUC, 36 suffered benign upper urinary tract lesions, and 24 had invasive RCC of the renal pelvis. Stratified by surgical modality, 144 patients were indicated for RUN, and 47 for KSS. After the secondary screening for UTUC patients, 95 patients were classified as MIUTUC, 60 as NMIUTUC. Our research process is displayed in Fig. 1.

### The role of urine FISH in surgical decision-making for upper urinary tract lesions

**Fig. 2:**
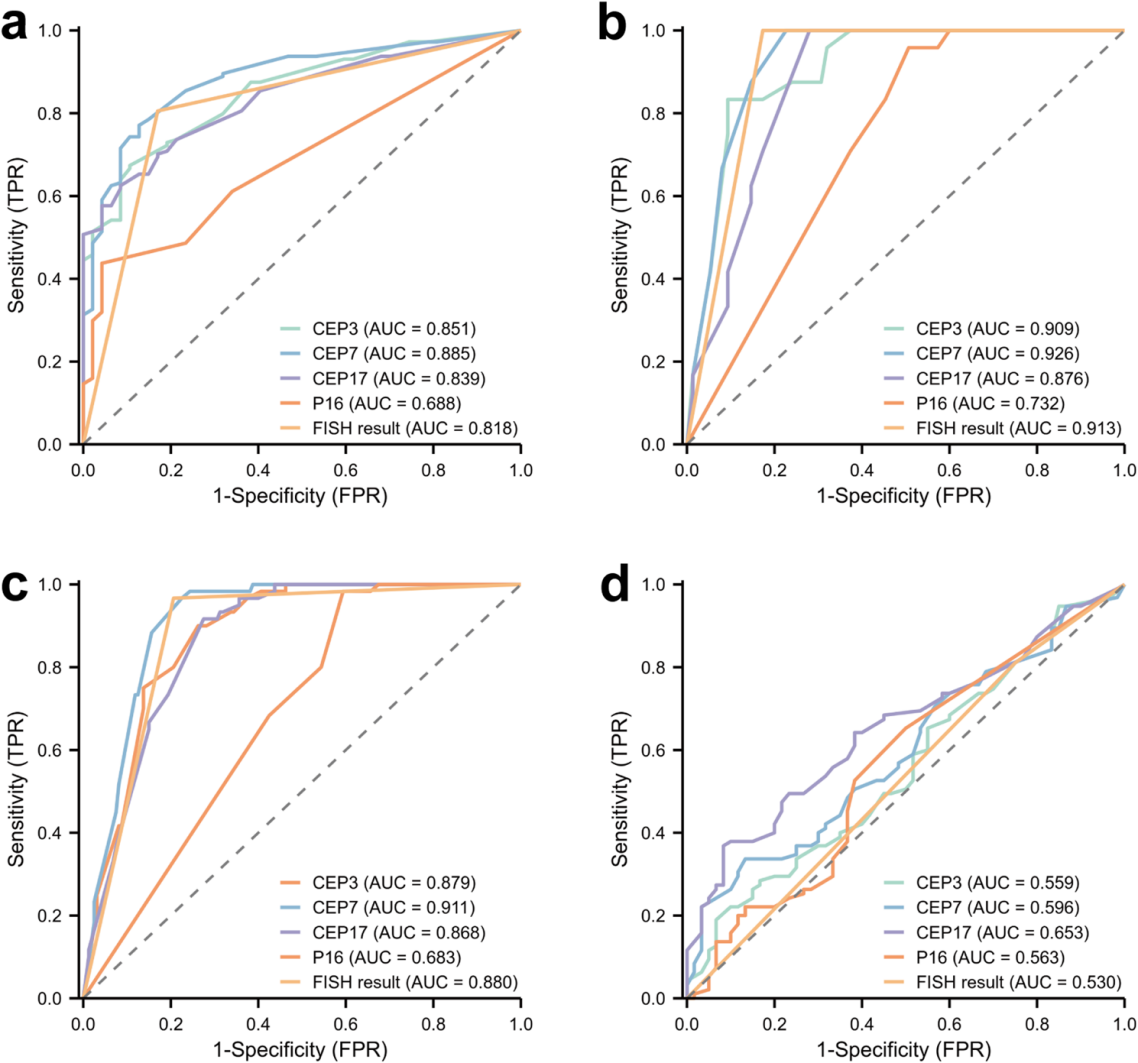
The extended clinical value of urine FISH in UTUC. **a** The ROC curves of urine FISH-guided surgical decision-making for upper urinary tract lesions. **b** The ROC curves of urine FISH for differential diagnosis of renal pelvis carcinoma. **c** The ROC curves of urine FISH for discriminating non-UTUC lesions. **d** The ROC curves of subjects predicted by urine FISH for tumor stage

Figure 2a presented the ROC curve of urine FISH features for predicting patients who require RUN (RUN group, *N* = 144 vs. KSS group, *N* = 47). The results indicated that urine FISH test could provide valuable insights for surgical protocol development. Among these features, CEP7 demonstrated the best predictive efficacy (AUC = 0.885), P16 exhibited the highest specificity (0.957), and FISH result showed the highest sensitivity (0.806) .

### Differential diagnostic ability of urine FISH for RPC

Infiltrating renal pelvic RCC and RPC can sometimes be difficult to differentiate on CTU (Fig. S2). Figure 2b demonstrated the ROC curve of urine FISH features for the differential diagnosis of malignant tumors in the renal pelvis (RPC group, *N* = 75 vs. RCC group, *N* = 74). Among all FISH features, CEP7 had the highest predictive efficacy (AUC = 0.926), with sensitivity, specificity, PPV and NPV of 1, 0.773, 0.585 and 1, respectively.

### The discriminative capacity of urine FISH for distinguishing UTUC from non-UTUC lesions

Figure 2c depicted the discriminative performance of urine FISH for distinguishing non-UTUC lesions from UTUC (UTUC, *N* = 160 vs. non-UTUC, *N* = 80). Notably, CEP7 demonstrated the highest predictive efficacy (AUC = 0.911), reaffirming the exceptional diagnostic value in urothelial carcinoma.

### Predictive value of urine FISH for UTUC staging

Figure 2d illustrated the predictive ability of urine FISH test for tumor staging (NMIUTUC, *N* = 60 vs. MIUTUC, *N* = 95). Regrettably, CEP3 (AUC = 0.559), CEP7 (AUC = 0.596), CEP17 (AUC = 0.653), P16 (AUC = 0.563) and FISH result (AUC = 0.530) were unsatisfactory in predicting tumour staging.

### CEP17-based staging prediction tool for UTUC staging

We observed limited predictive value of FISH for UTUC staging. Given that the muscle-invasive status of a tumor is crucial for guiding NAC and assessing patient prognosis, we aimed to combine other variables to further improve predictive ability.

Prior investigations have demonstrated that preoperative blood routine examination indicators and inflammatory markers serve as potential predictors of pathological stage in UTUC [16, 17]. Given the established clinical predictive utility, health economic advantages, broad applicability, and translational potential of preoperative hematological markers, we collected blood routine examination indicators and derived seven inflammatory markers based on these variables. Subsequently, 155 UTUC patients were randomly split into training and test sets. Table 1 demonstrated the reasonableness of our dataset partitioning. After conducting feature selection on 28 blood-related features and 5 urine FISH features, 10 features were ultimately identified to develop the interpretable ML model. Among 8 ML models, SVM delivered the best predictive performance (AUC = 0.84). Thus, it was chosen as the optimal model. In addition, the sensitivity, specificity, PPV, NPV, recall, and precision of all models in both sets are presented (Table 2). DCA was employed to compare and evaluate the aforementioned 8 prediction models, aiming to determine their potential benefits in clinical applications and to assess whether using predictive models could lead to improved medical decision-making. The results demonstrated that in the test set, the DCA curve of the SVM model was higher than those of the other models across the majority of risk threshold intervals. This indicated that, within most threshold ranges, clinical decisions guided by the SVM model could yield a higher net benefit. Compared to current treatment strategies, the SVM model further reduced the incidence of both overtreatment and delayed intervention (Fig. S3).

**Table 1:** Baseline characteristics of patients in the training set and test set

Characteristics	Training set(*N* = 108)	Test set(*N* = 47)	*P* value
Age, n (%)			0.12
≥ 65	69 (44.5%)	36 (23.2%)	
<65	39 (25.2%)	11 (7.1%)	
Gender, n (%)			0.669
Male	66 (42.6%)	27 (17.4%)	
Female	42 (27.1%)	20 (12.9%)	
Smoking Status, n (%)			0.419
Current/Former	37 (23.9%)	13 (8.4%)	
Never	71 (45.8%)	34 (21.9%)	
Tumor Location, n (%)			0.852
Renal pelvis	50 (32.3%)	24 (15.5%)	
Ureter	55 (35.5%)	22 (14.2%)	
Both	3 (1.9%)	1 (0.6%)	
Pathological Grading, n (%)			0.931
High Grade	96 (61.9%)	42 (27.1%)	
Low Grade	12 (7.7%)	5 (3.2%)	
Muscle Invasive, n (%)			0.945
Yes	66 (42.6%)	29 (18.7%)	
No	42 (27.1%)	18 (11.6%)	

**Table 2:** Predictive performance of 8 ML models in training and test sets

Cohort	Models	Sensitivity	Specificity	PPV	NPV	Precision	Recall	AUC
Training set	DT	0.76	0.83	0.74	0.85	0.74	0.76	0.9
	XGB	0.93	0.56	0.57	0.93	0.57	0.93	0.82
	LR	0.81	0.64	0.59	0.84	0.59	0.81	0.78
	Enet	0.76	0.68	0.6	0.82	0.6	0.76	0.75
	RF	0.79	0.95	0.92	0.88	0.92	0.79	0.95
	SVM	0.76	0.71	0.63	0.82	0.63	0.76	0.76
	LGBM	0.93	0.95	0.93	0.95	0.93	0.93	0.98
	KNN	0.88	0.64	0.61	0.89	0.61	0.88	0.81
Test set	DT	0.5	0.86	0.69	0.74	0.69	0.5	0.79
	XGB	0.67	0.66	0.55	0.76	0.55	0.67	0.75
	LR	0.67	0.79	0.67	0.79	0.67	0.67	0.8
	Enet	0.5	0.79	0.6	0.72	0.6	0.5	0.79
	RF	0.28	0.63	0.67	0.63	0.63	0.28	0.81
	SVM	0.72	0.72	0.83	0.72	0.72	0.72	0.84
	LGBM	0.44	0.67	0.71	0.67	0.67	0.44	0.78
	KNN	0.67	0.67	0.79	0.67	0.67	0.67	0.81

**Fig. 3:**
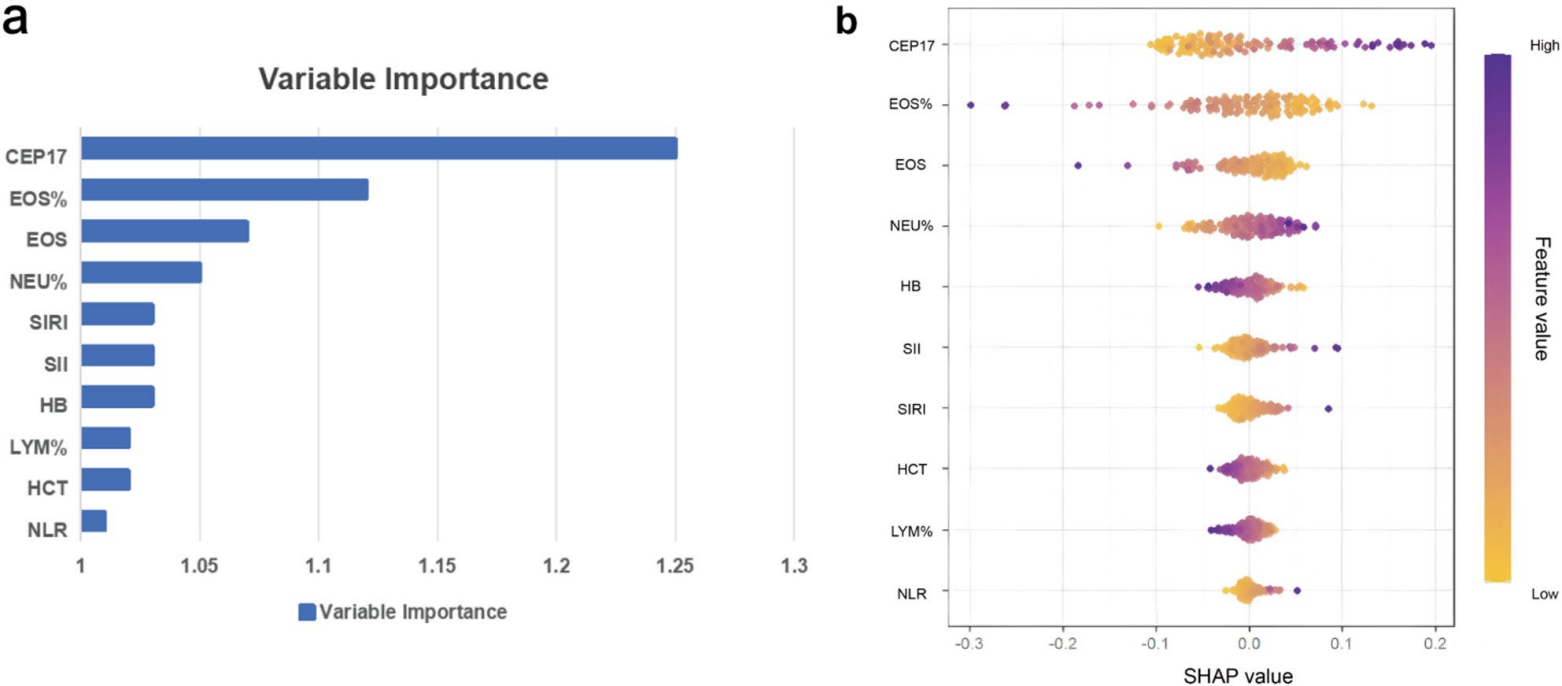
The interpretable SVM model. **a** The variable importance plot highlighted the ranking of incorporated feature importance. **b** The SHAP value plot described the directional influences of features on model predictions. EOS%: eosinophil ratio, EOS: eosinophil count, NEU%: neutrophil ratio, HB: haemoglobin, SII: systemic immuno-inflammatory index, SIRI: systemic inflammatory response index, HCT: hematocrit, LYM%:lymphocyte ratio, NLR: neutrophil to lymphocyte ratio

We calculated the SHAP values for each feature and found that CEP17 remained the most important contributor in predicting muscle invasion in UTUC (Fig. 3a and b).

### Other applications of urine FISH in UTUC

**Fig. 4:**
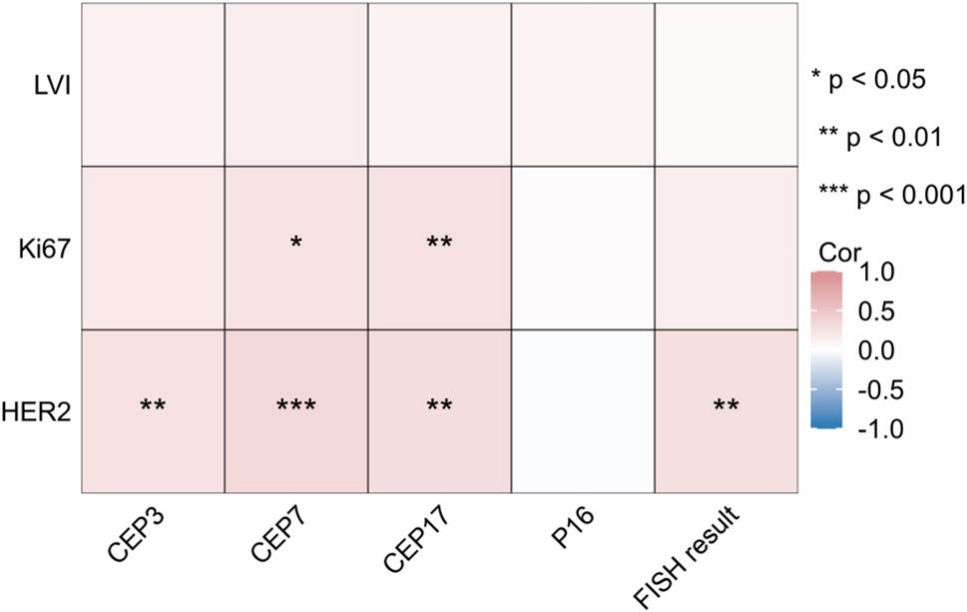
Heatmap of urine FISH features correlation with LVI, Ki67 and HER2

We analyzed the correlations of urine FISH features with LVI, Ki67, and HER2 (Fig. 4, Table S2). Spearman correlation analysis suggested that CEP7 and CEP17 were positively correlated with Ki67. CEP3, CEP7, CEP17, and FISH result were positively correlated with the level of HER2 expression (*P* < 0.01). However, the study failed to discover any correlation between urine FISH features and LVI.

## Discussion

Urine FISH has become an extremely important screening tool for UTUC [14]. Previous studies confirmed that urine FISH demonstrated notable advantages in the early detection of UTUC [18]. During the development of urothelial carcinoma, alterations in the P16 gene and chromosomes 3, 7, and 17 frequently occur [19]. In particular, deletion of the P16 locus, an oncogene, tends to indicate a higher degree of malignancy and a more aggressive tumour [20]. Aneuploidy changes in chromosomes 3, 7 and 17 are likewise associated with the malignancy of the tumour [21]. Thus, employing urine FISH to identify these specific abnormalities not only facilitates early detection but also offers vital information for tumor staging, grading, and differential diagnosis. In our study, we found CEP7 played a crucial role in optimizing UTUC surgical protocols and differentiating malignant tumors in the renal pelvic region. Although these features were ineffective in assessing tumour staging, integrating blood-related features and CEP17 markedly enhanced prediction power, providing a multidimensional molecular basis for UTUC precision medicine.

High-risk UTUC usually has a poor prognosis. In the absence of perioperative treatment, the 5-year survival rate is < 50% for patients with pT2/pT3 tumors and < 10% for patients with pT4 tumors [22]. These findings highlight the necessity for additional systemic therapy to enhance cancer control and improve patient outcomes. A review of 21 previous studies (involving 1983 patients) that confirmed the feasibility and clinical value of NAC prior to RNU [23]. However, due to the rarity of this disease and the limitations of preoperative testing, daunting challenges remain in screening patients for NAC [9, 10]. While Abdulrahman developed a predictive model based on enhanced CT for tumor staging in UTUC, the frequent comorbidity of UTUC with chronic renal disease and the renal burden imposed by contrast agents constrain the model’s practical applicability [24]. In this study, we successfully established a predictive tool applicable to most UTUC patients. It accurately predicted tumor stage only using preoperative urine and blood samples. Based on this model prediction, if patients are identified as having a high risk of muscle-invasive tumors, NAC is recommended to achieve complete or partial pathological remission preoperatively and thereby improve their prognosis. In addition, our data also indicate that patients with higher CEP3, CEP7, and CEP17 are more strongly recommended to undergo RUN. This approach reduces the risk of undertreating high-risk patients and overtreating those with low-risk profiles or benign upper urinary tract lesions, enabling more precise and individualized treatment decisions.

Both RCC and RPC are malignant tumors located in the kidney. Nonetheless, there are absolutely discrepancies between them in surgical approaches and NAC [12, 25, 26]. Misdiagnosing RPC as RCC may subject patients to the distress of a second surgery [27]. Typical cases of RCC and RPC can usually be easily distinguished through CT. Unfortunately, sometimes RCCs may grow into the renal sinus and invade the renal pelvis, sharing imaging similarities with RPC [8, 27]. Ultrasound-guided renal biopsy is effective for distinguishing these conditions but is less commonly used due to its invasive nature [28]. We propose urine FISH as a novel, noninvasive method for identifying tumor cell types at the genetic and chromosomal levels.

LVI and Ki67 are well-established histological surrogates of tumour aggressiveness, HER2 has also been validated as a pivotal therapeutic target for antibody–drug conjugates in UTUC. Although our data reveals only weak correlations between urine FISH features and these markers, this negative finding carries important implications. First, it underscores the necessity for future studies to enlarge sample sizes or integrate higher-dimensional molecular profiles to enhance statistical power; second, it sets a preliminary boundary on the independent predictive utility of urine FISH features in this context. Moreover, with the rapid evolution of machine-learning methodologies, the construction of multi-omics predictive models has become central to precision oncology. Systematically evaluating the associations between urine FISH features and LVI, Ki67, and HER2 provides a foundational data resource for identifying candidate predictors in future models.

Our study is constrained by several limitations. Firstly, due to its retrospective nature and single-center design, inherent bias is inevitable. Moreover, the rarity of UTUC has led to relatively small sample sizes in the included studies, potentially affecting data accuracy and the generalizability of findings. Additionally, our cohort accrued mainly between 2022 and 2025. Over half underwent surgery after 2024. The resulting short follow-up precludes reliable recurrence, progression, or survival analyses. Finally, despite the multiple measures taken to mitigate overfitting risk, this model must undergo rigorous external validation before clinical implementation.

Despite these problems, our findings hold prominent clinical implications. This study initially validated urine FISH features as a reliable evaluation marker, proving it to be a valuable adjunct in the surgical planning of upper urinary tract lesions. The prediction model exhibited robust performance, offering a promising reference for both the implementation of NAC and the assessment of patient prognosis. We still need to conduct larger, multi-center prospective studies to further validate our analysis.

## Conclusions

The clinical utility of urine FISH in UTUC likely transcends beyond mere diagnostic confirmation. Preoperative urine FISH analysis provides ancillary evidence for differential diagnosis, staging, and surgical decision-making, thus refining the precision of UTUC management.

## Supplementary Information

Supplementary Material 1.

## Data Availability

The datasets used and/or analysed during the current study available from the corresponding author on reasonable request.

## Abbreviations

AUA: American Urological Association
CEP3: Chromosome Enumerating Probe 3
CEP7: Chromosome Enumerating Probe 7
CEP17: Chromosome Enumerating Probe 17
CTU: Computed tomography urography
DCA: Decision curve analysis
DT: Decision tree
Enet: Elastic net
FISH: Fluorescence in situ hybridization
KNN: K-nearest neighbours
KSS: Kidney-sparing surgery
LGBM: Light gradient boosting machine
LR: Logistic regression
LVI: Lymphovascular invasion
MIUTUC: Muscle-invasive upper tract urothelial carcinoma
ML: Machine learning
NMIUTUC: Non muscle-invasive upper tract urothelial carcinoma
NPV: Negative predictive value
P16: P16 probe
PPV: Positive predictive value
RCC: Renal cell carcinoma
RF: Random forest
ROC: Receiver operating characteristic
RPC: Renal pelvis cancer
RUN: Radical nephroureterectomy
SVM: Support vector machine
UTUC: Upper tract urothelial carcinoma
XGB: Xgboost
