# Supplementary material for: Beyond diagnosis: exploring the extended clinical utility of urine fluorescence in situ hybridization in upper tract urothelial carcinoma
Source: BMC Urol. 2025 Dec 22;25:307. doi: 10.1186/s12894-025-02025-w (PMC12751602; doi:10.1186/s12894-025-02025-w)
Supplement: Supplementary file 1 — Supplementary Material 1. [file 12894_2025_2025_MOESM1_ESM.docx]

**Additional Section1**

**Appendix A. Criteria for determining FISH result**

For each sample, a minimum of 100 cells exhibiting consistent staining, non-overlapping nuclei, and distinct signals were analyzed for each probe. Diploid normal cells typically display two red and two green fluorescent signals when labeled with probes and observed under a fluorescence microscope. Aneuploidy or gene deletions manifest as deviations from this pattern, with corresponding increases or decreases in fluorescent signals. Evaluation criteria are based on the presence of abnormal signal quantities surpassing predefined thresholds for each probe.

1. The threshold of CEP3,CEP7 and CEP17 is 10%, and that of P16 is 15%;
2. When the abnormal signal exceeds the threshold value, the corresponding urine FISH feature is classified as abnormal;
3. FISH result is considered positive if two or more FISH features are found to be abnormal; otherwise, the result is considered negative.

**Appendix B. Grouping criteria**

Based on pathology findings, preoperative evaluations, and electronic health records, participants were categorized into distinct cohorts for further investigation into the efficacy of FISH in guiding surgical decision-making, distinguishing RPC, and forecasting UTUC staging. The delineated groups were as follows:

1. RUN group: RUN is the established treatment protocol for high-risk UTUC, defined by meeting any of the following criteria: 1) presence of multiple lesions; 2) lesion diameter ≥2 cm; 3) high-grade findings in urine cytology examination; 4) high-grade findings in biopsy; 5) evidence of imaging infiltration.
2. KSS group: KSS is the recommended approach for patients with low-risk UTUC, while lesion resection is advised for benign lesions in the upper urinary tract. These patient cohorts are categorized as nephron-sparing surgery group due to the availability of kidney-preserving surgical interventions. The diagnostic criteria for the low-risk UTUC include the following: 1) presence of a single lesion; 2) lesion diameter <2 cm; 3) low-grade findings in urine cytology examination; 4) low-grade findings in biopsy; 5) absence of imaging evidence of invasion. A diagnosis of low-risk UTUC can only be established when all criteria are met concurrently.

The diagnostic criteria for benign lesions of the upper urinary tract are as follows: Pathological diagnosis of benign lesions in the renal pelvis or ureter, including ureteral polyps, precancerous lesion, renal lipoma, renal angioleiomyolipoma, etc.

1. RPC group: The patient received a pathological diagnosis of urothelial carcinoma, with the lesion located in the renal pelvis.
2. RCC group: Pathological diagnosis of RCC and imaging shows tumor invasion of renal pelvis and calices. Such patients are easily confused with RPC, leading to an increased risk of overtreatment.
3. UTUC group: Pathologically confirmed UTUC, encompassing both renal pelvic and ureteral malignancies.
4. Non-UTUC group: Pathologically confirmed malignancies of non-urothelial origin or benign lesions.
5. NMIUTUC group: T staging of UTUC is Tis or Ta or T1.
6. MIUTUC group: T staging of UTUC is T2 or T3 or T4.

**Additional section2**

**Appendix C. Supplementary figure**


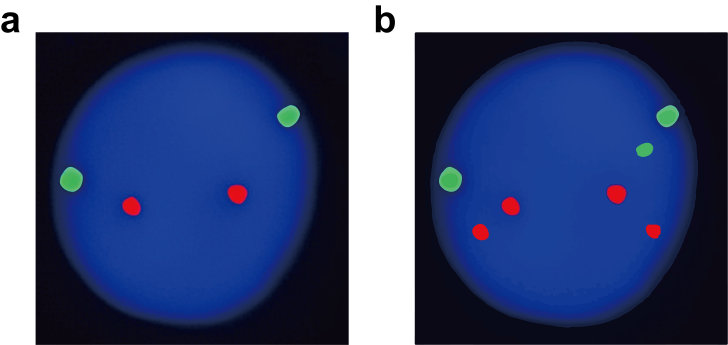


**Fig. S1** Schematic diagram of urine FISH test under fluorescence microscope (a) Microscopic manifestations of FISH in normal cell. (b) Microscopic manifestations of FISH in tumor cell


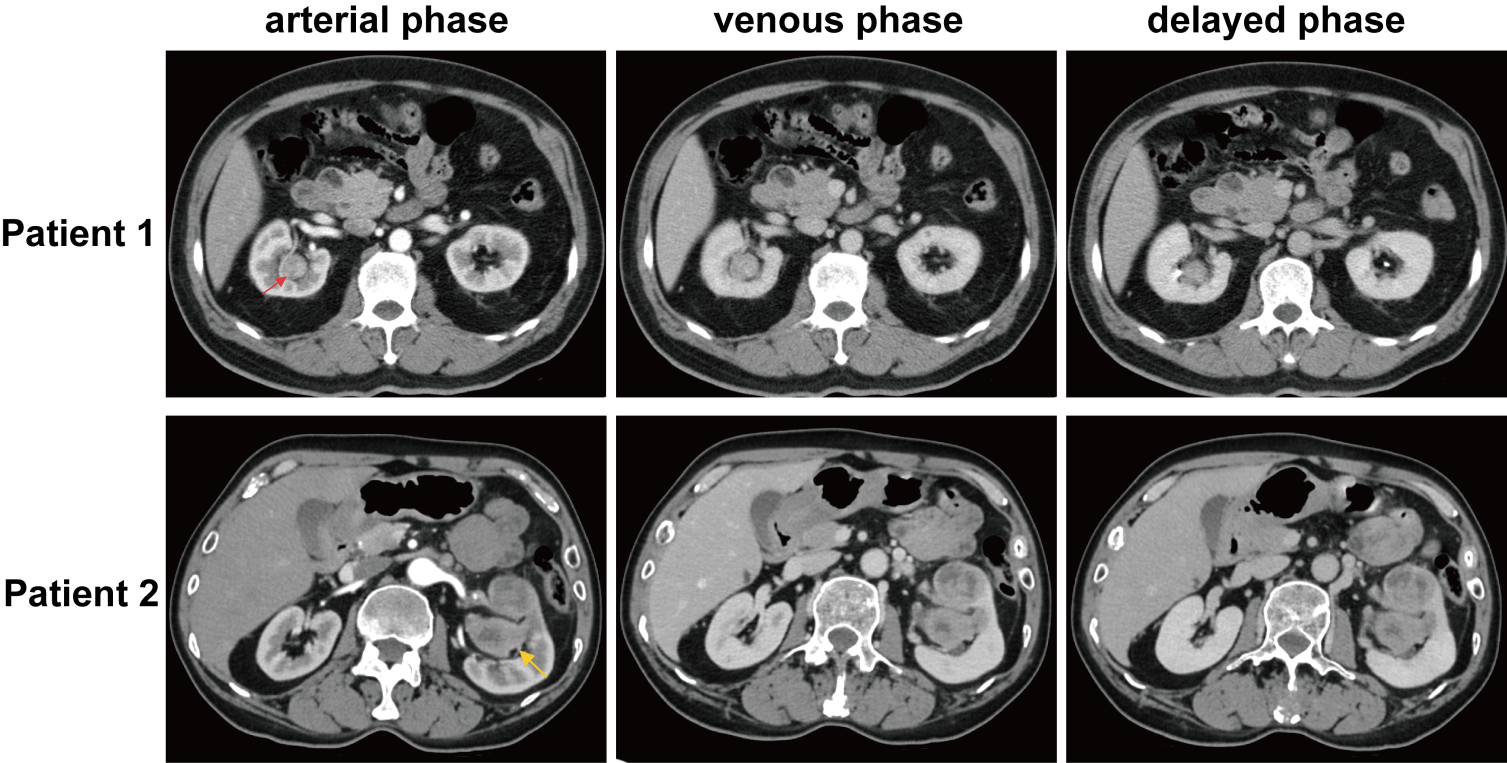


**Fig. S2** Infiltrating renal pelvic RCC and RPC axial CTU images. The red arrow indicates infiltrating renal pelvic RCC, and the yellow arrow indicates RPC


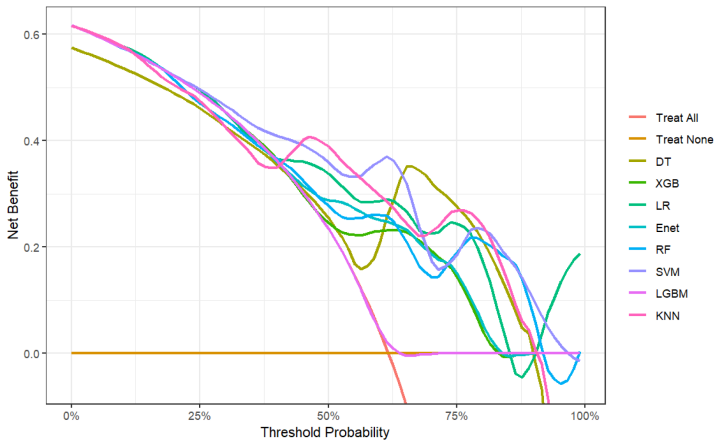


**Fig. S3** DCA decision curves of 8 ML models in the test set

**Additional section3**

**Appendix D. Supplementary table**

| **Table S1** All predictor variables for patients with UTUC | | |
| --- | --- | --- |
|  | *Predictor variable* | *Abbreviation* |
| Urine FISH features | **Chromosome Enumerating Probe 3** | CEP3 |
|  | **Chromosome Enumerating Probe 7** | CEP7 |
|  | **Chromosome Enumerating Probe 17** | CEP17 |
|  | P16 Probe | P16 |
|  | Fluorescence in situ hybridisation result | FISH result |
| Hematologic parameters | White blood cell count | WBC |
|  | Neutrophil ratio | NEU% |
|  | Lymphocyte ratio | LYM% |
|  | Eosinophil ratio | EOS% |
|  | Basophil ratio | BAS% |
|  | Monocyte ratio | MON% |
|  | Neutrophil count | NEU |
|  | Lymphocyte count | LYM |
|  | Eosinophil count | EOS |
|  | Basophil count | BAS |
|  | Monocyte count | MON |
|  | Red blood cell count | RBC |
|  | Haemoglobin | HB |
|  | Hematocrit | HCT |
|  | Mean Corpuscular Volume | MCV |
|  | Mean corpuscular hemoglobin concentration | MCHC |
|  | Red blood cell distribution width | RDW |
|  | Platelet count | PLT |
|  | Platelet distribution width | PDW |
|  | Mean platelet volume | MPV |
|  | Thrombocytocrit | PCT |
| Inflammatory indicators | Systemic immune inflammation index | SII |
|  | Platelet lymphocyte ratio | PLR |
|  | Neutrophil lymphocyte ratio | NLR |
|  | Lymphocyte monocyte ratio | LMR |
|  | Platelet neutrophil ratio | PNR |
|  | Systemic inflammatory response index | SIRI |
|  | White blood cell mean platelet volume ratio | WMR |

| **Table S2** Spearman correlation analysis between FISH features and LVI, Ki67, and HER2 | | | | | |
| --- | --- | --- | --- | --- | --- |
|  | CEP3 | CEP7 | CEP17 | P16 | FISH result |
| LVI | 0.11 | 0.15 | 0.12 | 0.2 | 0.06 |
| Ki67 | 0.19 | 0.24 | 0.26 | 0.03 | 0.15 |
| HER2 | 0.25 | 0.34 | 0.29 | -0.02 | 0.27 |
